# An overview of pharmacological activities of baicalin and its aglycone baicalein: New insights into molecular mechanisms and signaling pathways 

**DOI:** 10.22038/IJBMS.2022.60380.13381

**Published:** 2022-01

**Authors:** Zhihua Hu, Yurong Guan, Wanying Hu, Zhiyong Xu, Muhammad Ishfaq

**Affiliations:** 1 College of Computer Science, Huanggang Normal University, Huanggang 438000, China; 2 College of Veterinary Medicine, Northeast Agricultural University, 600 Changjiang Road, Xiangfang District, Harbin 150030, P. R. China; 3 Hubei Zhiying Medical Imaging Center, Radiology Department of Huanggang Hospital of Traditional Chinese Medicine, China; # These authors contributed equally to this work

**Keywords:** Baicalein, Baicalin, Cancer, Inflammatory diseases, Mitochondrial functions

## Abstract

The flavonoids, baicalin, and its aglycone baicalein possess multi-fold therapeutic properties and are mainly found in the roots of *Oroxylum indicum* (L.) Kurz and *Scutellaria baicalensis* Georgi. These flavonoids have been reported to possess various pharmacological properties, including antibacterial, antiviral, anticancer, anticonvulsant, anti-oxidant, hepatoprotective, and neuroprotective effects. The pharmacological properties of baicalin and baicalein are due to their abilities to scavenge reactive oxygen species (ROS) and interaction with various signaling molecules associated with apoptosis, inflammation, autophagy, cell cycle, mitochondrial dynamics, and cytoprotection. In this review, we summarized the molecular mechanisms underlying the chemopreventive and chemotherapeutic applications of baicalin and baicalein in the treatment of cancer and inflammatory diseases. In addition, the preventive effects of baicalin and baicalein on mitochondrial dynamics and functions were highlighted with a particular emphasis on their anti-oxidative and cytoprotective properties. The current review highlights could be useful for future prospective studies to further improve the pharmacological applications of baicalein and baicalin. These studies should define the threshold for optimal drug exposure, dose optimization and focus on therapeutic drug monitoring, objective disease markers, and baicalin/baicalein drug levels.

## Introduction

**Figure 1:**
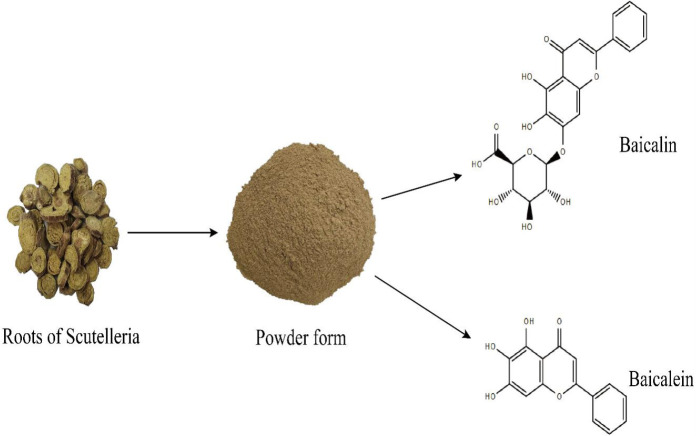
Roots of Scutellaria baicalensis, powder form, and chemical structure of baicalin and baicalein

The flavonoids, baicalin, and its aglycone, baicalein, were extracted from *S. baicalensis* Georgi (SBG), *Scutellaria galericulata*, *Scutellaria rivularia *Wall, and *Scutellaria lateflora* L. as well as in *O. indicum* (L.) Kurz (OI, Bignoniaceae) (1-6). *O. indicum* is mostly found in India, Sri Lanka, Pakistan, Bangladesh, Cambodia, China, Thailand, and other south Asian countries (7). Whereas, plant species of *Scutellaria* are grown in eastern Russia, Japan, China, Siberia, Mongolia, and Korea (8). The chemical structures of baicalin and baicalein are represented in Figure 1. Previous studies reported that the roots, seeds, and stem-bark of *O. indicum* have been applied for the treatment of diseases in China, India, and several other countries for the cure of dysentery, rheumatic pain, diarrhea, pharyngitis, coughs, and other respiratory diseases such as bronchitis, etc. (9). Besides, *Scutellaria radix* is used for the cure of dysentery, atherosclerosis, hyperlipidemia, hypertension, and respiratory ailments in traditional Chinese medicines (10). Baicalin and baicalein have been receiving much interest from cosmetic, food, and pharmaceutical industries due to their excellent antioxidant, anti-inflammatory, anticancer, antidiabetic, anti-ulcerative colitis, antithrombotic, antiviral, eye-protective, cardioprotective, neuroprotective, and hepatoprotective properties (11, 12). In addition, baicalin and baicalein exhibited anti-cancer and favorable against various anti-inflammatory disorders targeting relevant signaling pathways (13). Previous studies on baicalin and baicalein have summarized the pharmacological activities including anti-inflammatory, anti-tumor, cardioprotective, neuroprotective, anti-ocular disorders, and mitochondrial functions (14-16). However, limited information is available about the clinical uses and dose optimization of both compounds. In this review, we highlighted the signaling pathways of baicalin and baicalein in the treatment and chemoprevention anti-cancer, anti-inflammatory, antioxidative and cytoprotective properties as well as baicalin-baicalein’s interactions. This study could be useful in exploring the therapeutic drug targets, defining the threshold for optimal drug exposure, and dose-optimization that could lay a foundation to further enhance the biological activities of both compounds in curing various health disorders. 

***Protective effects of baicalin and baicalein against inflammatory disorders***

**Figure 2:**
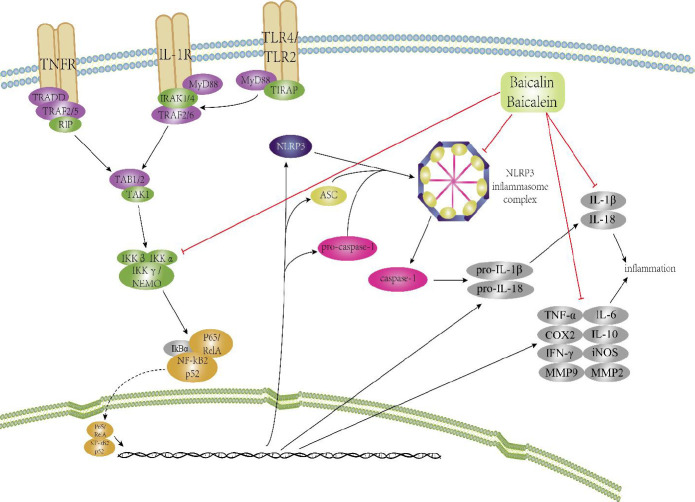
Effect and mechanism of baicalin and baicalein for inflammation-related ailments via inflammation-related signaling pathways are shown. Baicalin/baicalein effectively inhibited inflammation through NF-κB and inflammasome pathway

Inflammation is a protective reaction in a localized area and characterized by symptoms such are pain, redness, heat, loss of function, and swelling. Stimulation of IKKβ and IKKα and translocation of NF-κB to the nucleus leads to increased pro-inflammatory, anti-inflammatory cytokines, and chemokines to defend against shock or trauma (17). Chronic inflammation is often related to increased expression of NF-κB by the invaded microbes and injured tissues, leading to several diseases including asthma, cancer, inflammatory bowel disease, cardiovascular diseases, sepsis, psoriasis, atherosclerosis, rheumatoid arthritis, acquired immunodeficiency disorder syndrome (AIDS), gastritis, CNS depression, multiple sclerosis (MS), etc. (18-20). In the last couple of years, new genome-wide association studies have been carried out on common inflammation-related diseases; for instance, diabetes, asthma, rheumatoid arthritis, atherosclerosis, colorectal cancer, Crohn’s disease, and MS to determine alleles for these ailments (21-23), which drew the interest of researchers, because significant economic losses occurred due to these diseases (24). The effects of flavonoids for alleviation of inflammation-related ailments through inflammatory cytokines are discussed in this review. The mechanisms of baicalein and baicalin are depicted in Figure 2.

*Anti-inflammatory effects in respiratory ailments*

Recently, it has been demonstrated that baicalein given orally at doses of 50 and 100 mg/kg/d for 28 days significantly ameliorated pulmonary fibrosis in rats. The results indicated that baicalein significantly reduced the expressions of smad2/3 and TGFβ1 and markedly reduced miR-21 expression and expression of alpha-smooth muscle actin (α-SMA) and hydroxyproline content. It has been noted that baicalein exerts protective effects through the TGF-β/smad signaling pathway (25). Besides, baicalin at a dose of 120 mg/kg/d (IP) treatment for 28 days protected mice by decreasing collagen deposition, lung coefficient, and hydroxyproline levels through the ERK1/2 pathway. Thus, these outcomes unveiled that baicalin exhibited an antifibrotic effect possibly via adenosine A2a receptor, which is involved in tissue repair and inflammation process (26, 27). Baicalin pretreatment (30 mg/kg, IP) ameliorated heart dysfunction and pulmonary artery hypertension (PAH) by attenuating the elevated expression of p38 MAPK in tissue homogenates and reducing matrix metalloproteinase-9 expression in lung arterioles. Moreover, baicalin inhibited the elevated levels of cytokines via the p38 MAPK signaling pathway in the lung tissues (28). The inhibition of smooth muscle cells in the pulmonary artery offers treatment for PAH (29). Baicalin administration at doses of 10 and 20 mM/L significantly down-regulated p-Akt and HIF-1α protein and mRNA levels and elevated p27 proteins in rat PASMCs during hypoxia. In another *in vivo* study, baicalin (100 mg/kg, IP) significantly alleviated the right/left ventricle plus septum ratio and right ventricular systolic pressure (RVSP) via up-regulating p27 protein and suppressing the expression of mRNA expressions of p-Akt and HIF-1α via attenuating the increased Akt protein level (30). A previous study demonstrated that the human mast cells played a crucial role in respiratory disorders and were deposited at inflammatory sites in asthmatic (31) and allergic rhinitis patients (32). HMCs activate innate immune responses through secretion of IL-6, MCP-1, and IL-8 (33). In previous experiments, baicalein was reported to exhibit potential therapeutic effects in allergic and asthmatic disorders and inhibited IL-8, IL-6, and MCP-1 in the culture of the HMCs through degradation of IkBα and inhibition of both IkBα phosphorylation and NF-κB activation (34). 

*Anti-inflammatory effects in arthritis*

Inflammation of the joints, which results in joint destruction and bone and cartilage erosion is known as rheumatoid arthritis (RA) (35). Baicalin acts as a potential remedy for RA; in an *in vivo* study, baicalin (100 mg/kg/d, IP for 7 days) markedly reduced ankle swelling and inhibited splenic Th17 cell population in murine arthritic mice 14 days postimmunization. *In vitro* results revealed that baicalin (dose of 20 mM for 24 hr) inhibited IL-17-induced inflammatory cascade, blocked lymphocytes’ attachment to synovial cells, and decreased the expression of IL-6, TNF-α, intercellular adhesion molecule-1 (ICAM-1), and vascular cell adhesion molecule-1 (VCAM-1) (36). Baicalein (20 mg/kg orally) exhibited preventive effects against food allergy in mice by alleviating the signs of anaphylaxis, diarrhea, and rectal temperature and through activation of B cells and reducing serum IgE levels, as well as by promoting the function of intestinal barrier via regulating tight junctions in Caco-2 epithelial cells. Thus, baicalein may be used as a healing agent for the cure of inflammatory bowel diseases (IBD) and food allergies (17, 37). Baicalin alleviated ulcerative colitis in mice which is a form of IBD. The underlying mechanism of prevention involves modulation of polarization of macrophages via suppression of protein known as interferon regulatory factor 5 (IRF5) and enhanced the expression of IRF4 expression in dextran sodium sulfate-induced colitis in mice. In *in vitro* experiments, baicalin modulated M1 macrophage polarization in LPS-stimulated murine macrophages and reduced the expression of IL-23, IRF5, and TNF-α protein expressions (38). A previous study reported the clinical efficacy of baicalin in patients with RA and coronary artery disease. The lipid profiles of coronary artery disease were reduced with 20 mg baicalin after 12-week oral administration, further showing the effectiveness of baicalin in curing coronary artery disease (39). Recently, Isola, *et al*. compared and scrutinized the effectiveness of a phytotherapeutic drug composed of herbal extracts including baicalin on postsurgical discomfort, and it was noticed that the herbal extract composed of baicalin decreased the severity of postoperative pain compared with ibuprofen and placebo (40).

*Anti-inflammatory effects in type-2 diabetes and obesity*

Baicalin could be used for the treatment of obesity in humans. Baicalin at a dose of 80 mg/kg/d (IP) significantly reduced the bodyweight of high-fat diet (HFD) rats. In addition, baicalin decreased the elevated level of free fatty acids (FFA), TNF-α, and serum cholesterol through stimulation of acetyl CoA-carboxylate and AMP-activated protein kinase (AMPK). In HepG2 cells, baicalin-treatment (5 and 10 mM/L) for 24 hr decreased lipid accumulation through activation of AMPK (41). Previous reports demonstrated that baicalein at doses of 0.25 and 0.5 g/kg/d for 5 weeks proved effective in type 2 diabetes (T2D) mice, and showed improved glucose tolerance, blood insulin levels, and hyperglycemia. It has also been noted that baicalein (5 mM) in human islets culture cells and insulin-secreting pancreatic INS382/13 cells significantly promoted viability and enhanced glucose-stimulated insulin secretion (GSIS) (42). Besides, a recent study reported that baicalin and baicalein enhanced mitochondrial function and viability via a cAMP-dependent pathway (43). In HFD-fed mice, baicalein (400 mg/kg/d) supplementation alleviated inflammation, obesity, hyperglycemia, insulin resistance, and hyperlipidemia in diabetic mice through activation of AMPK, which in turn suppressed the synthesis of fatty acids and cholesterol by reducing the transcription of fatty acid synthase and SREBP-1c and elevated the level of PPAR-α and its downstream genes that are involved in fatty acid oxidation. In another study, 90 mg/ml baicalein pretreatment of hepatocytes culture and 25 mM solution of baicalein in AMPKα_2_-stimulated mice in glucose significantly down-regulated ERK and p38 phosphorylation, which results in inhibition of the MAPKs signaling pathway (44, 45).

*Anti-inflammatory effects in cardiovascular ailments*

Researchers have demonstrated that baicalein could be used for treatment of cardiovascular ailments. Researchers reported that baicalein at 200 mg/kg/d (IP) treatment for 12 weeks ameliorated fibrosis of heart tissues in rats via suppression of p-ERK, 12-lipoxygenase, and matrix-metalloproteinase-9 (MMP-9) and alleviated the intraventricular septum thickness (46). Atherosclerosis, where arteries become hardened and narrowed, results in stroke, coronary thrombosis, myocardial infarction, and other cardiovascular-related ailments (47, 48). Baicalin/baicalein can be a promising agent for alleviating cardiovascular-related diseases. A previous report explained that baicalin and baicalein (5 and 10 mM) reduced vascular inflammation through suppression of disruption of endothelial barrier function and cellular adhesion molecules, and up-regulation of NF-κB in cultured human umbilical vein endothelial cells (HUVECs) (49). In vascular smooth muscle cells (VSMCs) of rats, baicalin (20, 40, and 60 mM) for 24 hr significantly reduced the migration and proliferation of VSMCs by enhancing the expression of the p27 protein and reducing the level of E-CDK2 protein. Moreover, treatment with baicalin at a dose of 70 mg/kg/d in rats significantly reduced the thickness of the left carotid artery by inhibiting neointimal hyperplasia through suppression of the protein cyclin E (PCNA) and up-regulation of p27 proteins level (50). Baicalin administration (20 mg/kg, IV) markedly restored vascular function through suppression of the NF-κB pathway and plasma superoxide anions, iNOS, NO, and TNF-α by improving blood pressure in LPS-induced septic rats (51). Studies indicated that baicalein suppressed NF-κB, which in turn inhibited thioredoxin reductase (TrxR) activity in lymphocytes (52). Similarly, another researcher reported that baicalein at a dose of 10 mg/kg (IV) reduced myocardial inflammatory responses, apoptosis, and oxidative stress through suppression of iNOS, MCP-1, cardiac superoxide anion, phospho-p65, and phospho-IkBα proteins in LPS-induced septic rats (53, 54). In experimental autoimmune encephalomyelitis (EAE) mice, baicalin at doses of (5 and 10)-mg/kg/d, (IP) significantly reduced the severity of the disease according to scoring (2.2+0.3), induced IL-4 expression, suppressed IFN-γ, and inhibited mononuclear cell proliferation (55). In an EAE model of mice, baicalin at a dose of 100 mg/kg/d (IP) for 20 days ameliorated the severity of the disease by reducing immune cell infiltration through up-regulating cytokine signaling-3 (SOC-3) protein and inhibited Th-17 and Th-1 cell differentiation (56). 

*Anti-inflammatory effects in liver diseases*

Baicalein acts as an effective therapeutic strategy for autoimmune hepatitis (AIH). Baicalein at a dose of 10- and 20-mM induced apoptosis through mitochondrial pathway in concanavalin A-stimulated CD3^+^ T cells. Previously, it has been shown that baicalein (100 mg/kg, IV) alleviated liver injury in mice through suppression of IFN-γ and TNF-α (57). Researchers reported that the prolonged use of baicalein at a dose of 40 and 80 mg/kg/d orally for 10 weeks alleviated CCl_4_-induced liver fibrosis in rats. The mechanisms involved inhibition of hepatic stellate cell activation and reduced ALT, AST, laminin, collagens, and hyaluronic acid in the liver serum (58). Baicalein (80 mg/kg/d, orally) for 4 d significantly ameliorated CCl_4_-induced acute liver injury in mice through suppression of inflammatory cytokines IL-6 and TNF-α (59). In another animal model, application of baicalin at a dose of 70 mg/kg/d (IP) for 56 days significantly reduced liver index, collagen deposition area, AST, ALT, IL-6, and TNF-α. In contrast, the IL-10 level was up-regulated in CCl_4_-induced liver fibrotic rats (60).

*Anti-inflammatory effects in neurodegenerative diseases*

Baicalein proved an effective anti-inflammatory mechanism against neurodegenerative diseases. Baicalein administration (560 and 280 mg/kg/d) protected mice neurotoxicity caused by 1-methyl-4-phenyl- 1,2,3,6- tetrahydropyridine (MPTP) and reduced apoptosis by alleviating mitochondrial dysfunction of dopaminergic neurons (61). Another study also reported that baicalin (200 mg/kg/d) exerted preventive effects in MPTP-induced neurotoxicity in mice treated for a period of one week (62). In human SH-SY5Y cells, baicalein (50 and 100 mM) for 24 hr significantly reduced ROS production, attenuated apoptosis through Bcl-2 and Bax proteins, and inactivated the ERK1/2 pathway (63). Additionally, baicalein (0.5 and 5 mg/ml) for 24 hr attenuated apoptosis and alleviated parkinsonism in 6-hydroxydopamine (6-OHDA)-stimulated SH-SY5Y cells. In a rat 6-OHDA-induced parkinsonism model, baicalein given at a dose of 200 mg/kg/d for 15 days reduced apoptosis and down-regulated ROS of neurons (64). Previous studies demonstrated that baicalein could be used as an anti-inflammatory in chronic kidney diseases. In unilateral ureteral obstruction (UUO)-induced mice model of renal fibrosis, baicalein administered at a dose of 100 and 50 mg/kg/d for a week significantly reduced the accumulation of collagen and fibronectin through suppression of NF-κB expression and its downstream genes including IL-1β, TNF-α, and MCP-1 expression and inactivating the MAPK signaling pathway (65).

*Anti-inflammatory effects in microbial infections*

Recently, studies reported that baicalin acts as a potential anti-inflammatory agent against bacterial infections. For instance, *Mycoplasma gallisepticum* (MG) infection causes significant losses in the poultry industry (66, 67). Researchers reported that baicalin (50, 100, and 200 mg/kg BW) suppressed the NF-κB pathway through TLR4 receptor in LPS-induced infection in chicken liver. The underlying mechanism of action involves suppression of inflammatory markers including iNOS, IL-1β, COX-2, TNF-α, and IL-6 expression. The levels of AST, ALT, and NO content were significantly reduced compared with the control group (68). In a model of MG-infection, baicalin at a dose of 450 mg/kg attenuated oxidative stress and apoptosis in chicken spleen through elevation of Nrf2/HO-1 signaling pathway and suppressed the NF-κB pathway (66). Similarly, another study reported that baicalin (450 mg/kg) significantly ameliorated structural damage in the chicken thymus and alleviated MG-induced inflammatory cell infiltrates, reduced oxidative stress and expression of pro-inflammatory cytokines, and attenuated apoptosis (69). In addition, baicalin (450 mg/kg) administration for 7 days significantly reduced inflammation in chicken lungs and trachea during MG infection. The authors reported that baicalin treatment restored energy metabolism in chicken lungs and attenuated apoptosis through the mitochondrial pathway (70, 71). Most of the studies reported the molecular mechanism of both compounds, employed for the cure of diabetes, inflammatory bowel diseases, cardiovascular disorders, rheumatoid arthritis, respiratory ailments, kidney diseases, neurodegenerative diseases, hepatitis, cancers, and autoimmune encephalomyelitis. It is worthy to mention that several aspects of baicalein and baicalin such as bioavailability need to be studied before for the treatment of human ailments in clinical cases (72, 73).

***Protective effects of baicalin and baicalein on cancer***

**Figure 3:**
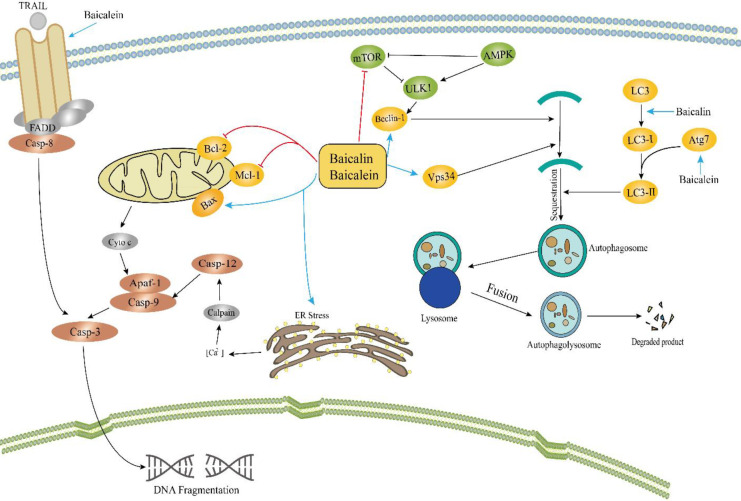
Baicalin and baicalein triggered programmed cell death in cancer cells via releasing cytochrome-C from mitochondria in the intrinsic pathway. Baicalein activated the TNFR-associated death domain (TRADD) through the extrinsic pathway. In addition, baicalin or baicalein activated autophagy in cancer cells and intervened in the formation of autophagosomes in various steps. The pathways involved the inhibition of AKT/mTOR and activation of AMPK/ULK1

Apoptosis is programmed cell death, triggered by two pathways: the extrinsic and intrinsic pathways (74). The intrinsic pathway (mitochondrial pathway) is often activated by viral infections, toxic materials, ionizing radiation, different cytokines, certain types of hormones, or loss of growth factors. The extrinsic pathway (receptor-mediated pathway) is often activated to exterminate unhealthy cells through activation of the death receptor located on the cell membrane (75, 76). Numerous studies reported that baicalin and baicalein induced apoptosis in various types of tumors through both extrinsic and intrinsic pathways (77). The anti-cancer effects of baicalin and baicalein are multi-fold, such as, through induction of apoptosis and triggering of autophagy as shown in Figure 3.

*Programmed cell death in cancer cells*

Baicalein mainly induced apoptosis through Ca^+2^ influx via Ca^2+^ release from the reticulum to cytosol dependent on phospholipase C protein. ROS production is associated with baicalein-induced apoptosis via Ca^2+^-dependent apoptosis in tongue and breast cancer cells (78, 79). An intracellular calcium chelator BAPTA was applied to inhibit caspase-3 activity to confirm that apoptosis was induced by baicalein in MDA MB-231 cells. The level of Bax/Bcl-2 increased and caspase-3 and -9 were activated following the release of cytochrome C (80). In gastric cancer cells, baicalein mediated apoptosis in a dose-dependent manner through disruption of mitochondrial membrane potential (81). It has been reported that baicalein forms hydrogen bonds with Asp253 and Ser251 at the active site of caspase 3 and interacts through its hydroxyl groups with Asp228 and Ser251 residues in caspase 9, which results in activation of these caspases (82). In pancreatic cancer cells, baicalein induced apoptosis via suppression of the Mcl-1 protein. In contrast, Mcl-1 protein overexpression significantly alleviated baicalein-induced apoptosis (83). Additionally, researchers reported several signaling pathways associated with baicalein/baicalin-induced apoptosis. In HepG2 cells, baicalin-copper induced apoptosis through down-regulation of phosphoinositide-3 kinase/protein kinase B/mammalian target of rapamycin (PI3K/Akt/mTOR) signaling pathway (84). Similarly, baicalin down-regulated the level of anti-apoptotic protein Bcl-2 and activated caspase-3 and caspase-9 in breast cancer cells through the ERK/p38 MAPK signaling pathway (85). Studies demonstrated that baicalein treatment suppressed Bad, ERK1/2 phosphorylation, and MEK1 expression both *in vitro* and *in vivo*. While baicalein-induced inhibition of human hepatocellular carcinoma was reversed by overexpression of MEK1 (86, 87). Baicalein enhanced the activity of death receptor-5 (DR5) in prostate cancer PC3 cells. It has been noted that DR5 was enhanced both at protein and mRNA levels (88). In herbal medicine, baicalin is the active ingredient that acts as Fas ligand and caused up-regulation of Fas protein (89). 

*Suppression of metastasis*

Baicalin/baicalein not only induced apoptosis in cancer cells but also suppressed metastasis. Tumor metastasis is a multistep process including invasion, survival, arrest, and colonization (90, 91). Previous studies demonstrated that both baicalin and baicalein inhibited epithelial-mesenchymal transition (EMT) through the suppression of TGF-β in breast epithelial cells through the NF-κB pathway (92). In another study, baicalein suppressed metastasis in gastric cancer through inactivation of the Smad4/TGF-β pathway (93). Baicalein down-regulated the Wnt/b-catenin pathway which results in the suppression of metastasis in breast cancer through suppression of EMT (94). Similarly, another study reported the same results: baicalein inhibited SATB in the MDA-MB-231 human breast cancer cell line (95). A number of studies investigated baicalin and baicalein inhibition of the expression level of matrix metalloproteinases (MMP) such as MMP-9 and MMP-2 in liver, breast, lung, ovarian, gastric, and colorectal cancers and glioma (96-103). Baicalein suppressed metastasis in prostate cancer cells via suppression of the caveolin-1/AKT/mTOR pathway (104). Besides, novel G protein-coupled estrogen receptor (GPR30) signaling pathway was inactivated by baicalein and decreased phosphorylation of Akt and ERK as well as tyrosine phosphorylation of epidermal growth factor receptor (EGFR) in breast cancer cells, leading to suppression of migration and invasion of cancer (105). Apart from these results, baicalein (2.5-40 mM) suppressed metastasis through inhibition of phos-Ezrin and total Ezrin protein, which plays a crucial role in tumor development (106). In addition, in gallbladder cancer, baicalein suppressed metastasis through Zinc finger protein, X-linked (ZFX) (107). Moreover, baicalein interferes with platelet aggregation involved in the early stage of metastasis and down-regulated the PI3K kinase activity (108). Baicalin and baicalein both have the potential to control angiogenesis through basic fibroblast growth factor (bFGF) (109, 110). In human umbilical vein endothelial cells (HUVECs), baicalein suppressed angiogenesis via the p53/Rb signaling pathway (111). Baicalin attenuated lung metastasis through inhibition of hypoxia-inducible factor (HIF) (112). Similarly, baicalein reduced the transcription activity of HIF, responsible for activating genes associated with angiogenesis such as vascular endothelial growth factor (VEGF) in MCF-7 cells (113). Baicalein acts as an anticancer agent via inhibiting 12-lipooxygenase (12-LOX), which produces 12(S)-hydroxy eicosatetraenoic acid (HETE), associated with tumor growth, proliferation, and metastasis (87, 114-117). 

*Triggering of autophagy and cell cycle arrest in cancer cells*

**Figure 4:**
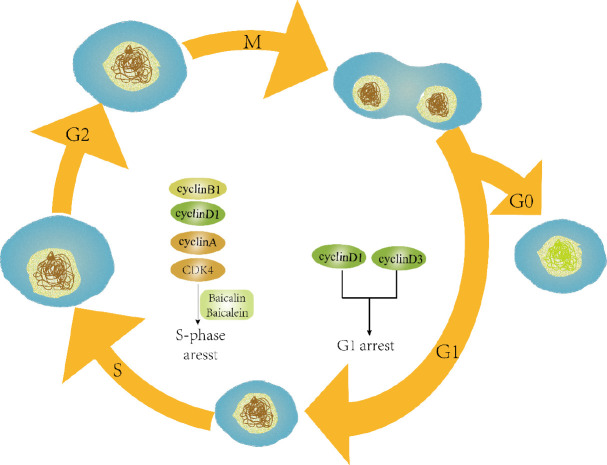
Baicalin or baicalein arrested the cell cycle at various checkpoints and caused inhibition of cyclin-D3 and cyclin-D1, which results in inhibition of the cell cycle at the G1 phase. The compounds, baicalin and/or baicalein also reduced the expression of cyclin-A, cyclin-B1, cyclin-D1, and CDK-4, leading to the arrest of the S phase in the cell cycle

It is well documented that autophagy is involved in the degradation of foreign invaders or dysfunctional cytoplasmic organelles by the lysosomes (118, 119). Studies reported that autophagy plays a critical role in the progression and inhibition of cancer (120, 121). Baicalin and baicalein induced autophagic cell death associated with several autophagy-related proteins as shown in Figure 3. In an *in vitro* assay, baicalin triggered autophagy in a dose and time-dependent manner through Beclin-1 and down-regulated CD147 (122). Similarly, both flavonoid compounds (baicalein and baicalin) caused autophagy in various cancers including cleavage of LC3, autophagic flux, autophagosome formation and activation of Atg5/Atg7, Beclin-1, and vacuolar protein sorting 34, involvement of AKT/mTOR pathway, and activation of RelB/p52 proteins (123-128). Besides apoptosis and autophagy, these compounds also induced cell cycle arrest at certain checkpoints in cells as shown in Figure 4. Baicalin and baicalein arrested the S phase in the cell cycle through suppression of cyclin A in lung cancer A549 cells and decreased the level of cyclin D1 in SK-MES-1 cells (129). In human lung squamous carcinoma CH27 cells, baicalein (50 mM) treatment for 24 hr induced cell cycle arrest at G0/G1 phase through down-regulating the expression of CDK4, cyclin D1, and cyclin B1 (130). Moreover, baicalein in combination with silymarin decreased cell growth in the S-phase along with increase in the G0/G1 phase in HepG2 cells, implicated in down-regulation of CDK4, cyclin D, phosphor-Rb, and cyclin E and up-regulation of p53, Rb, p27 (Kip1), and p21 (Cip1) (131). In HepG2 cells, a dramatic increase has been noticed in G2/M population, whereas in Hep3B cultured cells, a significant increase was observed in sub G1 (hypoploid peak) caused by baicalin and baicalein (132). In another study, G0/G1-phase arrest has been noted 12 hr post-treatment of baicalein and S-phase arrest in MCF-7 cells, showing that baicalein effectively inhibited cancer via cell cycle arrest at different phases (133). In hepatocellular carcinoma cells, baicalein induced G0/G1-phase arrest through inhibition of cyclin D1 and β-catenin pathway (134). Besides, it has been reported that baicalein induced G1 phase arrest and facilitated degradation of cyclin D1 through activation of aryl hydrocarbon receptor (AhR) (135). Therefore baicalin/baicalein could be efficiently used as a potential candidate against a variety of tumors. 

***Protective effects of baicalin and baicalein on mitochondrial function and interactions***

**Figure 5:**
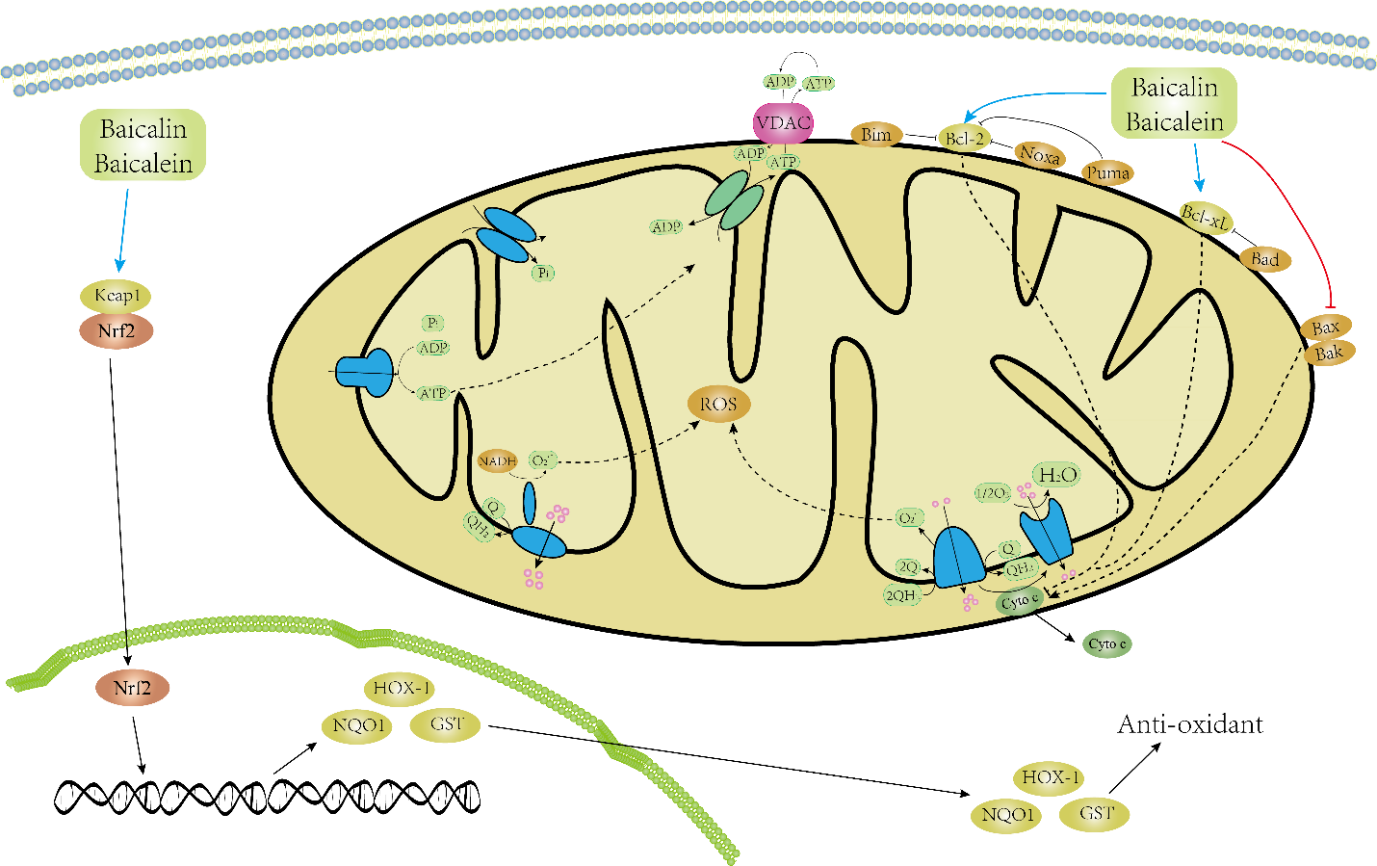
Baicalin or baicalein prevents mitochondrial dysfunction. Baicalein improves mitochondrial redox-related aspects and enhances mitochondrial activity. Baicalein reduces the changes in mitochondrial dynamics and loss of MMP. Moreover, baicalin/baicalein activates Nrf2, the master regulator of ROS and its downstream antioxidant genes, and therefore balances the redox system, improves mitochondrial function, and exerts cytoprotective effects

It is well understood that mitochondria are the powerhouses of the cells, which provide energy and play a crucial role in maintaining cell homeostasis and cell death (136-138). Oxygen (O_2_) is necessary for generation of energy in mitochondrial bioenergetic reactions (136, 138). ROS is produced because of electron transport chains such as superoxide anion and reactive oxygen free radical (139-142). Excessive ROS produced should be catalyzed to avoid damage to biomolecules including RNA, DNA, lipids, and proteins. Antioxidant enzymes such as glutathione (GSH), bioactive molecules, and vitamins protect the cells from free radicals and ROS-induced damage (143, 144). Mitochondria also play a crucial role in cell death during maintenance of tissue homeostasis and development through the intrinsic apoptotic pathway (145, 146). The mechanism of baicalin/baicalein in the protective role of mitochondria is still not completely understood. However, studies demonstrated that baicalin/baicalein protects mitochondrial dysfunction. The protective effects of baicalin/baicalein on mitochondria are shown in Figure 5.

*Protective effects of baicalein and baicalin on mitochondrial signaling pathways*

Baicalein (6.25 and 12.5 µM) improved cell viability and protected mitochondria against 6-hydroxydopamine (6-OHDA)-induced toxicity in SH-SY5Y neuroblastoma cells (147). In addition, baicalein pretreatment (2 hr) protected mitochondria through the redox-dependent mechanism in cellular toxicity caused by N-acetylcysteine (147). In PC12 cells, baicalein at a dose of 5-40 µM prevented MMP imbalance 12 hr post-treatment and reduced apoptosis through the reduction of Bax and increased Bcl2 contents (148). Moreover, in human epidermal melanocyte (PIG1) cells, pretreatment with baicalein (10-40 µM) for 1 hr reduced MMP loss, inhibited the activation of caspase, and reduced apoptosis in PIG1 cells exposed to H_2_O_2_. The underlying mechanism involved the stimulation of nuclear factor erythroid 2 [NF-E2]-related factor 2 (Nrf2) and inhibition of cytochrome c release from mitochondria in Chinese hamster lung fibroblast (V79-4) cells (149). A study reported that baicalein administration for 27 days at doses of 30 or 100 mg/kg inhibited the loss of MMP, improved the production of ATP, increased the consumption of ADP and respiration control ratio (RCR) in the rotenone rat model (150). In a model of benzo[a]pyrene-induced carcinogenesis, baicalein treatment (12 mg/kg) for 16 weeks restored the normal function of mitochondria via the suppression of ROS, reduced GSH content, and enzyme activities such as isocitrate dehydrogenase–ICDH, malate dehydrogenase-MDH, α-ketoglutarate dehydrogenase–α-KDH, succinate dehydrogenase-SDH, cytochrome c oxidase, and NADH dehydrogenase. The authors suggested that baicalein treatment contributed to the protection of mitochondrial function through the maintenance of bioenergetic reactions associated with the Krebs cycle (151). Baicalin administration (120 mg/kg) for 30 days attenuated streptozotocin-induced mitochondrial damage in a rat model of diabetes (152). Baicalin at a dose of 200 mg/kg protected mitochondria in a rat model of hepatic ischemia/reperfusion (I/R) (153). In addition, baicalin reduced oxidative stress and decreased inflammation through NF-κB suppression and its downstream genes and prevented mitochondrial swelling (66, 154). Baicalin (50 µg/ml) for 1 hr improved cell viability, decreased superoxide ion, ATP production, and inhibited loss of MMP. The data revealed that baicalin up-regulated mitochondrial biogenesis through peroxisome proliferator-activated receptor gamma coactivator 1-alpha (PGC-1α) by 40% during heart failure and hypertrophy (155). Yan and Liu studied the effect of baicalin (0.8–1.5 mM) on mitochondria isolated from the brain of rats. They found that baicalin decreased state 3 but did not affect MMP and state 4, and also did not prevent mitochondrial changes in hypoxic conditions (156). Numerous studies investigated the effect of baicalin/baicalein on cell signaling pathways associated with mitochondrial functions in the context of mitochondrial physiology maintenance and cell fate (157-163). A previous study demonstrated that the combination of the two flavonoids, baicalin, and baicalein produced a stronger effect on mitochondria in the process of apoptosis by activating caspase 3 and caspase 9 activation and triggering the release of cytochrome c (145, 158). As discussed in this section, baicalin and baicalein may exert an indirect role in preventing mitochondrial dysfunction through blocking mitochondrial ROS production and loss of MMP. Overall, the two flavonoids can increase ATP production in situations of stress and amplify mitochondrial functions.

*Baicalin-baicalein’s interaction and signaling pathways*

Despite extensive studies in several therapeutic areas owing to the antioxidant, anti-bacterial, anti-cancer, and anti-inflammatory properties of baicalin and baicalein; there is still limited information available about baicalin-baicalein’s interaction and their association with signaling pathways. Previously, Lai *et al*. demonstrated the pharmacokinetics/metabolism of baicalin and baicalein in rats. Following oral dosing of baicalin, extensive levels of baicalein conjugated with glucuronide and sulfate were observed in the systemic circulation. Besides, absorption of baicalein was negligible after oral administration in rats (164). Another study investigated that baicalin underwent extensive metabolism through conjugation reactions in the intestine of rats and rapidly converted to its aglycone baicalein. In addition, the metabolism of baicalin was affected by the higher loading dose which results in the saturation of metabolism reaction and surpassed the first-pass metabolism (165, 166). Liu *et al*. explained that the rapid conversion of baicalin conjugates to baicalein could be due to increased intestinal beta-glucuronidase activity due to the diabetic condition in rats (166, 167). Based on the evidence with regard to the metabolism of baicalin, there appears to be an imminent challenge for researchers to study the molecular mechanisms of baicalin and baicalein in association with cell signaling pathways. It is worth mentioning that cytochrome p450 (CYP) enzymes and their downstream signaling pathways are of prime importance in the context of baicalin-baicalein’s interactions. For example, a researcher demonstrated that daily administration of baicalin induced CYP2B6 in human subjects (168). While another study showed that baicalin acts as a hepatoprotective against acetaminophen-induced hepatic injury by inhibiting the expression of CYP2E1 (169). However, it also raises an important challenge if baicalin modulates the expression of CYP enzymes in clinical therapy. This may influence the metabolism of other drugs (170-172). Therefore, baicalin-baicalein’s interaction studies will further provide clarity of the signaling pathways that are associated with the mechanism of action of baicalin and baicalein. 

**Table 1:** Summary of the protective effects of baicalin/baicalein in different experimental models

S. No.	Experimental model	Mechanisms and associated signaling pathways	Dose	Ref.
1	Rat	Inhibited apoptosis by suppressing via mitochondrial signaling pathway	100 mg/kg	
2	Rats	Reduced intracellular calcium level and lactate dehydrogenase release.	0.35, 3.5, 10 and 35 pM	
3	Mouse	Protective effects on hepatocarcinogenicity	50 and 100 mg/kg	
4	Microglial cells (mice)	Inhibited NO production	0.1, 1, 10 and 50 pM	
5	Rat	Decreased mitochondrial swelling, NF-kB activation, and suppressed caspase activation	200 mg/kg	
6	Mouse	Protective effects on colon cancer	50, 100 and 200 mg/kg	
7	Microglial neurotoxicity	Suppressed iNOS expression, and inhibited the binding activity of transcription factors with DNA	1, 5, 10, 20 and 25 pM	
8	Culture of Neuron-glia extracted from the embryos of E-14 rat	Restored [3H] dopamine uptake and loss in tyrosine hydroxylase-immunoreactive neurons. Alleviated the increased expression of superoxide, NO and TNF-α	1, 5, and, 10 pM	
9	Mouse	Reducing gallbladder cancer	15, 30, and 60 mg/kg	
10	Culture of HT22 cell	Alleviated the iodoacetic acid (IAA)-induced toxicity in cells	1–10 pM	
11	Cell line (PC12 cells)	Suppressed ROS production in PC12 cell line	0.1, 1, and 10 pM	
12	Brain injury due to trauma	Reduced TNF-a, IL-6 protein, and mRNA expression	30 mg/kg, IP	
13	Rat diabetes model	Baicalin protected mitochondrial damage from STZ-induced morphological changes	120 mg/kg for 30 days	
14	Endothelial cells of the human brain	Inhibited the degradation of claudin-5 protein and protected endothelial cells	10 pM	
15	Mouse	Reducing cervical cancer	80 mg/kg	
16	SH-SY5Y and PCI2 cells	Ameliorated cell apoptosis and promoted neurite outgrowth	0.05, 0.5 and 5 pg/ml	
17	Mice pulmonary carcinogenesis model	Decreased the mitochondrial ROS production and protected mitochondrial damage	12.0 mg/kg once a week	
18	Rat CCR model	Improved mitochondrial integrity by reducing MMP	30 or 100 mg/kg.day-1	
19	Mouse	Inhibited prostate cancer	10, 20, and 40 mg/kg	
20	Cell culture of COS-7 cells	Up-regulated TREK-2 protein in a direct/indirect manner	100 pM	
21	Culture of CATH.a cells	Up-regulated the intracellular GSH content and inhibited the dopamine quinone formation	1 pM	
22	Mouse	Prevent lung cancer	12 mg/kg	
23	Rat	Increased phosphorylation of Akt and CREB and inhibited LTP potentiation	0.1, 1, 10, and 50 pM	
24	Culture of PC12 cells line	Inhibited apoptosis and stimulated Nrf2/HO-1 pathway	50, 100, and 200 pM	
25	Rotenone-induced neurotoxicity in PCI2 cells	Inhibited ROS, apoptosis, and caspase 3/7 activation in PCI2 cells	10, 20 and 40 pM - PC12 cells.0.5 and 5 pM mitochondria.	
26	Mouse	Proved effective against pancreatic cancer	1% S. baicalensis diet	
27	Rat	Inhibited hepatic cancer	250 mg/kg	
28	Culture of PCI2 cells line	Suppressed the A|3-induced cytotoxicity and A|5 aggregation	0.1, 1, and 10 pM	
29	Mouse	Reduced prostate cancer	10, 20, and 40 mg/kg	
30	Culture of SH-SY5Y cells	Suppressed ROS and NO inside the cells and reduced extracellular NO production	0.02, 0.2, and 2 pM	
31	SK-N-MC cells	Modulated caspase-9 and Bax activities and bcl2 proteins	10, 20, 40, and 50 pM	
32	Mouse	Protected against bladder cancer	0.8 mg/mouse	
33	Mouse	Inhibited mucoepidermoid cancer effects	50, 100, and 200	
34	Culture of rat cortical neurons and astrocytes	Protected neurons through production of VEGF and Epo expression in neurons	3.5, 10, and 35 pM	
35	Culture of CHO cells	Suppressed the production of A|5 and increased APP a-secretase.	2.5, 5, and 10 pM	
36	Mouse	Proved effective against skin cancer	1 mg/cm2 skin area/mouse/100 ml acetone	
37	Culture of cortical neurons of mice	Protected neurons from cell death	30 pM	
38	Culture of cortical neurons of rats	Inhibited dopaminergic neuron loss, suppressed up-regulation of JNK and ERK, and prevented the translocation of NF-kB to the nucleus	10 pM	
39	Culture of SK-N-SH and SH-SY5Y cells	Prevented mitochondrial dysfunction and suppressed ROS production.	10, 40, and 80 pM	
40	Culture of cortical neurons	Enhanced sodium current and protected ROS	1 nM-10 pM	
41	Culture of rat hippocampal cells	Suppressed glutamate release via regulating depolarization	5-70 pM	
42 31	Culture of glial cells (C6)	Suppressed the generation of H2O2 and ROS, and protected mitochondrial integrity	0, 25, 50 and 100 μM	
43	Culture of microglial cells	Inhibited iNOS protein expression and NO production through down-regulating TLR4	0.1, 1, 10 μM	
44	Culture of cortical neurons of mice	Inhibited the depolarization caused by Aβ/AMPA/NMDA	4, 8, and 14 μM	
45	Mice model	Restored LIMK1, SNCA, and GLRA1 expressions to normal and protected the behavior of mice	140 and 280 mg/kg	
46	Rat model	Inhibited p-GSK3|3 protein, up-regulated p-Akt and p-PI3 K. Inhibited apoptosis through reducing the expression of caspase-3 and caspase-9.	2 and 4 mg/kg	
47	Rat model	Reduced the expression TLR4 and NF-kB translocation to the nucleus.	30 and 100 mg/kg	
48	Collagenase-induced ICH rat model	Increased ZO-1 protein expression and reduced iNOS protein. Inhibited the phosphorylation of JNK and p-38 MAPK and suppressed the NF-kB pathway.	15 and 30 mg/kg	
49	MPTP-induced neurotoxicity in Zebrafish	Reversed locomotor deficiency and prevented dopaminergic loss in neurons	10, 20, and 50 pM	
50	Mouse	Inhibited pancreatic cancer	1% in diet	1

## Conclusion

Abundant scientific evidence revealed that flavonoid compounds have protective effects on cancer, inflammation, and act as potential anti-bacterial, anti-viral, and antioxidants (as summarized in Table 1). Among them, baicalin and baicalein received much attention from researchers. In this review, ample evidence has been shown from previous studies that baicalin/baicalein has the potential to mitigate oxidative stress injury, alleviate inflammation, and combat tumors in various experimental models. In addition, baicalin/baicalein improved mitochondrial functions through redox-dependent mechanisms. However, there are still limited clinical studies on baicalin/baicalein. Most of the previous studies worked on the molecular mechanisms of the two compounds (11, 173, 174). Therefore, it is still difficult to undertake a clear decision on the use of the two natural compounds and their clinical impacts. Future studies are needed to enhance the bioavailability of baicalin and baicalein including nano-emulsion, solid-liquid nanoparticles, nano-crystallization, and baicalin/baicalein-loaded liposomes. Researchers should work in different experimental models on non-toxic doses to avoid toxicity, side effects, and adverse reactions. Modern approaches such as transcriptomics, system biology, and metabolomics are needed to perform to find its therapeutic targets, optimize dosage and enhance its bioavailability through different routes. The synergism/antagonism of baicalin/baicalein in combination with other drugs is still not reported in detail. Another important issue is the interaction studies of baicalin and baicalein in modulating several signaling pathways at multiple areas including absorption, distribution, metabolism, and excretion. Hence, the clinical settings of these phytomedicines including their long-term toxicity study, pharmacokinetics, and pharmacodynamics are to be carried out by different routes to obtain maximum efficacy in various ailments. 

## Authors’ Contributions

ZH and MI Supervised the research; MI, WH and YG Draft manuscript preparation; ZX Critically revised the paper. ZH, MI, WH, YG, and ZX Read and approved the final version to be published.

## Conflicts of Interest

We confirm that none of the authors have any competing interests.
